# Supplementary material for: Electrically driven cation exchange for in situ fabrication of individual nanostructures
Source: Nat Commun. 2017 Apr 12;8:14889. doi: 10.1038/ncomms14889 (PMC5394283; doi:10.1038/ncomms14889)
Supplement: Supplementary Information — Supplementary Figures, Supplementary Tables and Supplementary References. [file ncomms14889-s1.pdf]

Supplementary Figures

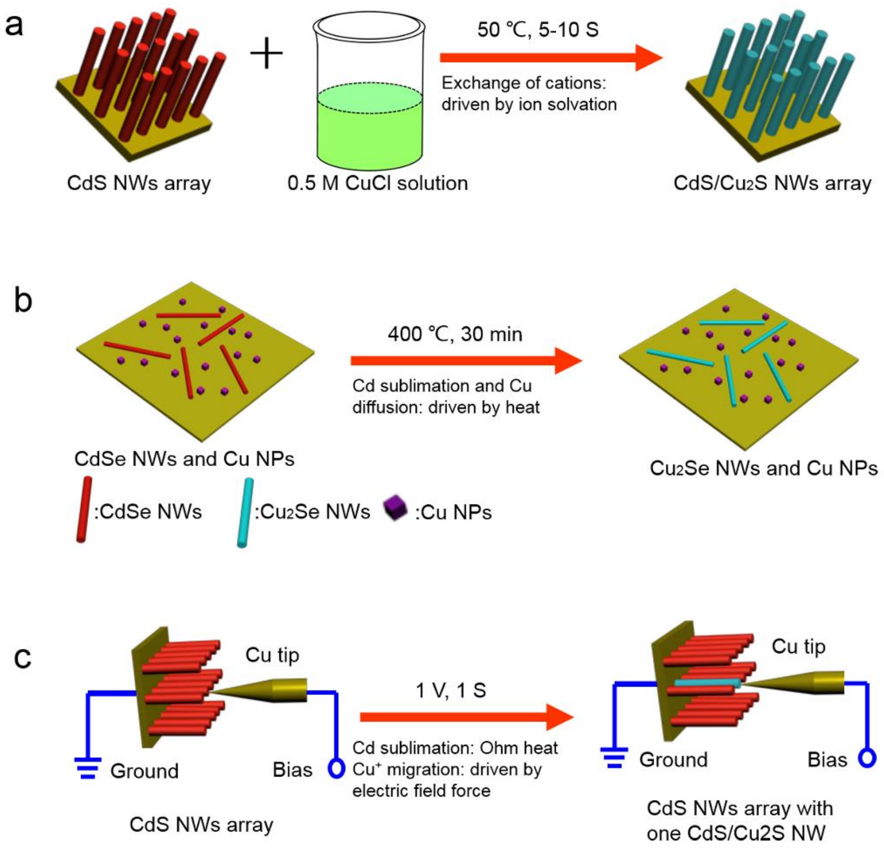

Supplementary Figure 1| The three types of cation exchange (CE) methods. a, Schematic diagram of the CE reaction in solution. The CdS nanowires (NWs) array was dipped into 0.5 M CuCl solution at 50 ℃ for 5-10 s, washed with deionized water, acetone and isopropanol, and dried under blowing nitrogen[1]. The exchange of Cd^{2+} and Cu^{+} is activated by the ion solvation[2,3]. **b**, Schematic diagram of the thermally activated CE reaction. CdSe NWs precursor and Cu nanocrystals as the local copper source deposited on an amorphous solid substrate. After thermal treatment at 400 ℃ for 30 min, the CdSe NWs were transformed into Cu_2Se NWs, through the complete sublimation of Cd and the insertion of Cu atoms[4]. Both the sublimation of Cd and the in-diffusion of Cu are driven by heat. **c**, Schematic diagram of an electrically activated CE reaction. The CdS NWs array is placed on an inert metal substrate and the Cu tip acts as the copper cations source. The Cu tip can move so as to contact any individual CdS nanowire (NW) in the array. After biasing with 1 V for 1s, the CdS NW has transformed into a CdS/Cu_2S core-shell structure. The sublimation of Cd is driven by Ohmic heating and the migration of Cu^{+} is driven by electric field force.

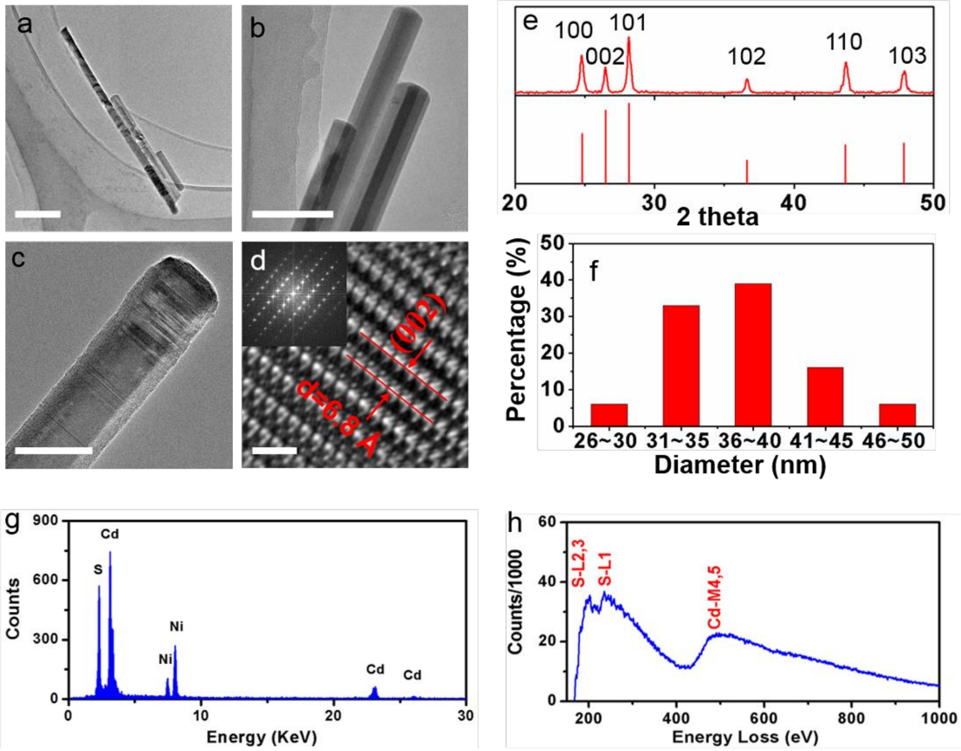

Supplementary Figure 2| Structural characterization of CdS NWs. a-b, Representative transmission electron microscopy (TEM) images of as-grown CdS NWs with length ~1μm (**a**) and with diameter ~40 nm (**b**). **c**, TEM image of an individual CdS NW. **d**, High-resolution transmission electron microscopy (HRTEM) image of the CdS NW showing single crystal. Inset: FFT image of the HRTEM, showing NW with <001> growth direction. **e**, Experimental (top) and reference (bottom) XRD spectrum of CdS NWs. **f**, The diameter distribution of the CdS NWs. **g**, EDX spectrum of CdS NWs. The presence of Ni is attributed to the Ni grid support. **h**, EELS spectrum of CdS NWs. The scale bars for (**a**), (**b**), (**c**) and (**d**) are 200 nm, 100nm, 50 nm and 2 nm.

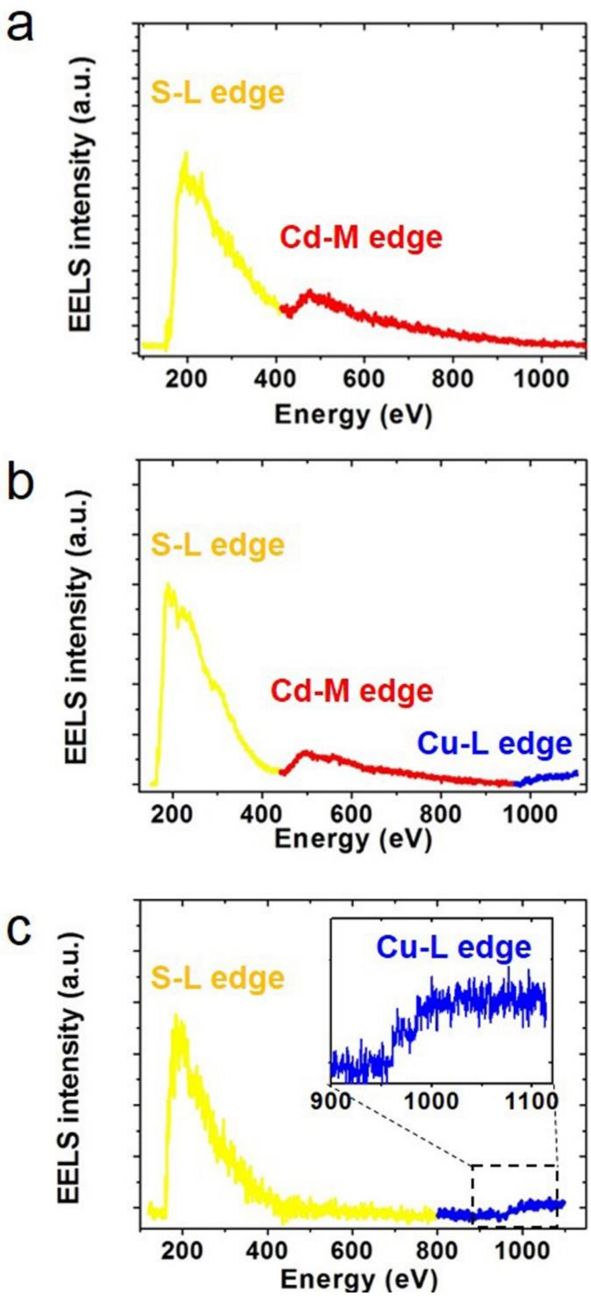

Supplementary Figure 3| Representative EELS spectra corresponding to the elemental mapping TEM images. The representative EELS spectra from (**a**) the CdS NW in Figure 1b, (b) the core and (c) the shell of the Cu_2S/CdS core shell structure NW in Figure 1g.

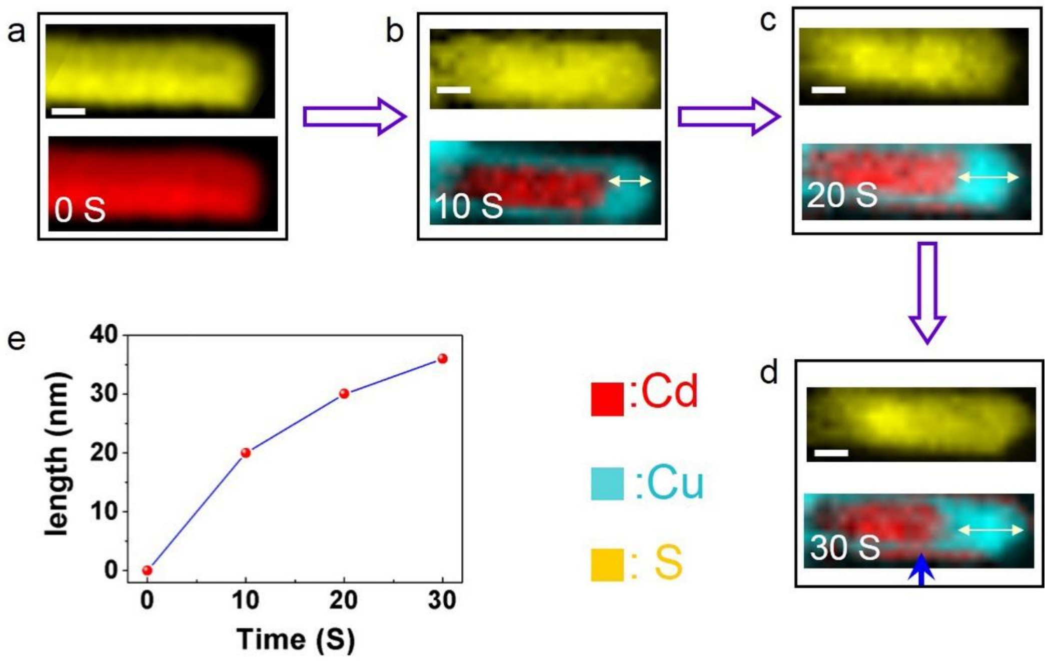

Supplementary Figure 4| Controlled growth of CdS/Cu_2S core-shell structured NWs. a-d, The NW was biased with a 0.5 V positive voltage, and the EELS mapping pictures were recorded at 0 s (**a**), 10 s (**b**), 20 s (**c**) and 30 s (**d**). Scale bar, 20 nm. The white two-way arrows mark the length of the top part (Cu_2S) of the NW at different times. **e**, The line plot of length vs. time illustrates that Cu_2S growth rate reduces with the increasing of biasing time.

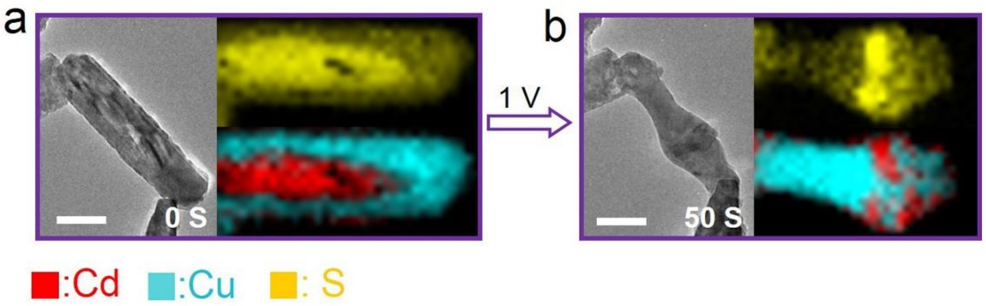

Supplementary Figure 5| Further biasing leading to the deformation of a partially cation-exchanged NW. **a**, TEM image and EELS mappings showing the NW with a core-shell structure. **b**, Further biasing of 1 V for 50 s, the deformation of CdS/Cu_2S NW was observed. Scale bar, 50 nm.

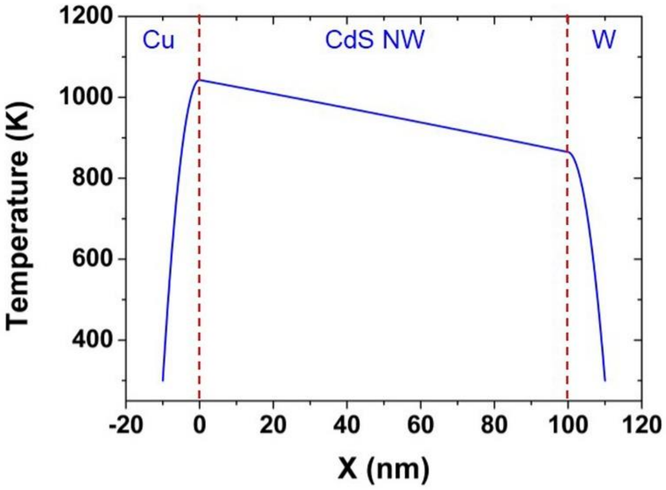

Supplementary Figure 6| Result of finite element method (FEM) simulation showing temperature distribution from the Cu electrode through the CdS NW to W tip.

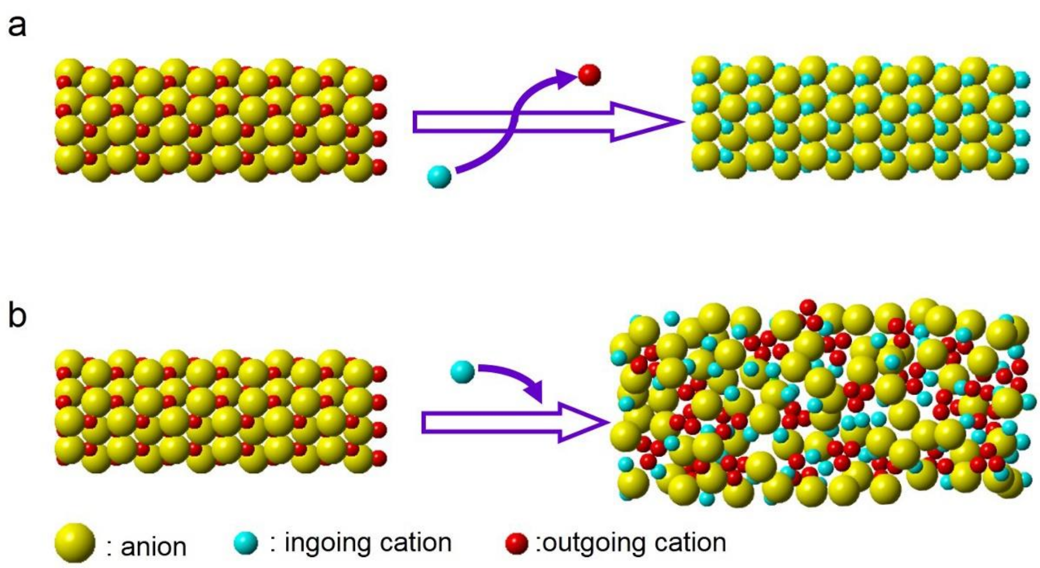

Supplementary Figure 7| CE reaction mechanism compared to materials conversion mechanism during the lithiation of anodes. a, Schematic diagram of CE mechanism. In this reaction, outgoing cations in precursor are replaced by inwardly migrating cations, after which they sublimate into the gas phase or dissolve in the solvent. The anion sublattice remains intact during the whole process which ensures that the initial shape and size can be maintained[5]. **b**, Schematic diagram of materials conversion mechanism during the lithiation of anodes. In this replacement reaction, the ingoing and outgoing cations in trade places to form a new element and a new compound but the outgoing cations won't be disappeared and they still existed in the internal or on the surface of the compound. The anion sublattice is also destroyed and therefore, the size and the shape cannot be preserved[6].

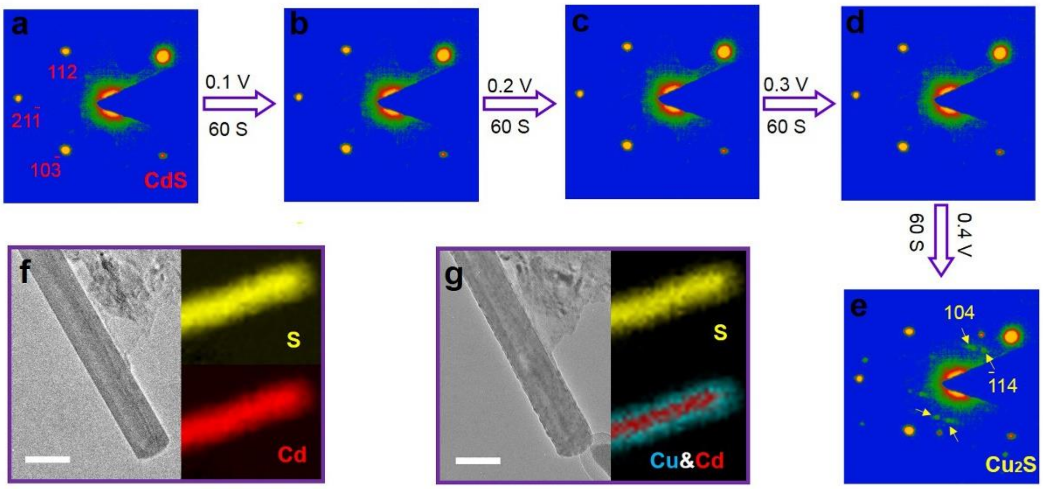

Supplementary Figure 8| Influence of applied electrochemical potential on the feasibility of electrically driven CE reaction. a, The SAED pattern of the pristine CdS NW. **b-e**, The SAED patterns of NW after 60 S biasing with different voltages (0.1 V (**b**), 0.2 V (**c**), 0.3 V(**d**) and 0.4 V(**e**)). **f**, The TEM image and corresponding EELS mappings of the pristine CdS NW. **g**, The TEM image and corresponding EELS mappings of the NW after biasing of 0.4 V for 60 S, which indicates the CE reaction is feasible. Scale bar, 50 nm.

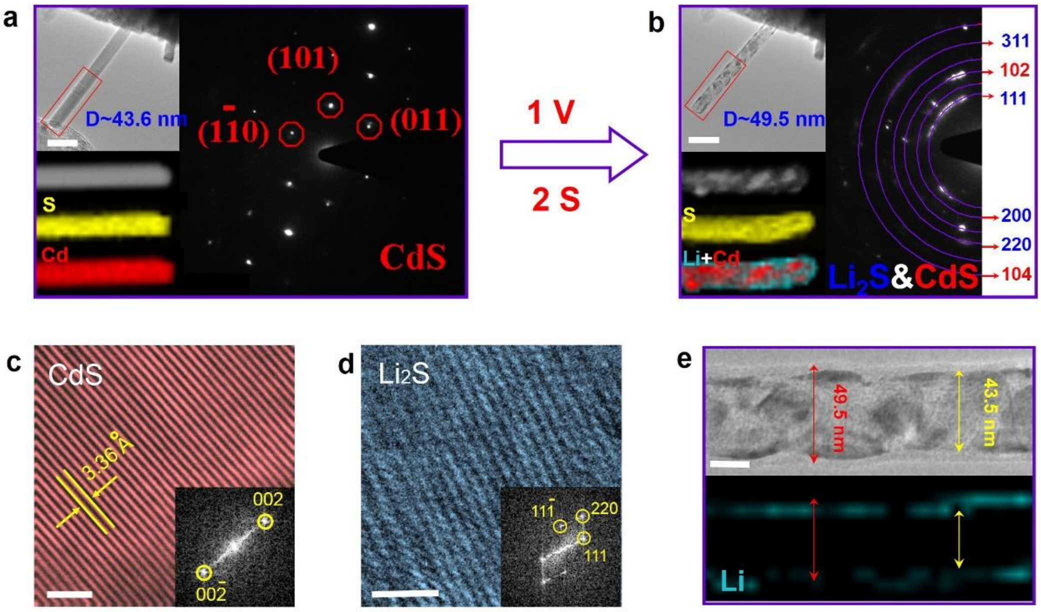

Supplementary Figure 9| TEM characterization of a single CdS NW before and after reaction with lithium. a, Image of a single CdS NW along with the associated SAED pattern, STEM picture and EELS mapping images (sulfur element mapping (yellow) and cadmium element mapping (red)) before biasing. The pictures show CdS NW with the hexagonal structure. Scale bar, 100 nm. **b**, Image of the NW along with the associated diffraction pattern, STEM picture and EELS mapping images (sulfur element mapping (yellow), cadmium element mapping (red) and lithium element mapping (indigo)) after biasing. The diffraction pattern confirms that the NW after biasing is composed of wurtzite CdS and cubic Li_2S. The EELS mapping images show that part of the Cd element is replaced by Li element. Scale bar, 100 nm. **c**, HRTEM image and the corresponding FFT of the selected CdS region in the NW after biasing. Scale bar, 2 nm. **d**, HRTEM image and the corresponding FFT of the selected Li_2S region in the NW after biasing. Scale bar, 2 nm. **e**, Top: TEM image of Li/Li_2S/CdS heterostructure. Scale bar, 20 nm. The outer diameter is 49.5 nm and the inner diameter is 43.5 nm. Bottom: EELS mapping data of the outer most shell in the top image indicating that the outer most shell is made of lithium. Noticeably, in order to get the elementary composition of shell, this bottom EELS mapping image is obtained using the Li element map minus the S element map, which means there is only Li element existing in shell.

Supplementary Tables

Supplementary Table 1| Crystal structure data for CdS[7,8] and the different phases of $Cu_{2-x}S$[8-10]

	Composition	Crystal Structure		
		Hexagonal		
**CdS**	CdS	a_1=4.160 Å	c_1=6.756 Å	
		Hexagonal		
**High-chalcocite**	Cu_2S	a_2=3.95 Å	c_2=6.75 Å	
		Monoclinic ($48Cu_2S$)		
**Low-chalcocite**	$Cu_{1.997-2}S$	a_3=15.246 Å	b_3=11.884 Å	c_3=13.494 Å
		β_3=116.35°		
		Monoclinic ($48Cu_{31}S_{16}$)		
**Djurleite**	$Cu_{1.93-1.97}S$	a_4=26.897 Å	b_4=15.745 Å	c_4=13.494 Å
		β_4=90.13°		
		Triclinic ($4Cu_{29}S_{16}$)		
**Roxbyite**	$Cu_{1.74-1.82}S$	a_5=13.409 Å	b_5=13.405 Å	c_5=15.485 Å
		α_5=90.022°	β_5=90.021°	γ_5=90.022°

Supplementary Table 2| Reaction type, reaction equation, dominant driving force and the role of electricity in different electrically activated processes[6,11-18]

	Reaction Type	Reaction Equation	Dominant Driving Force	The role of electricity
**Electrically driven cation exchange**	Electrochemical: replacement reaction	$2e^{-}+2M^{+}+CdS \rightarrow M_2S+Cd$	Electric field force	Driving the reaction
**Electromigration in metal**	No	No	Electronic wind force	Driving atoms migration
**Phase transformation in individual nanocrystal**	Chemical: replacement reaction	$2yLi+M_xS_y \rightarrow yLi_2S+xM$	Strong reducibility of lithium	Bring the lithium atom to nanocrystals
**Lithiation of anode (intercalation/de-intercalation materials)**	Electrochemical: combination reaction	$e^{-}+M^{+}+X \rightarrow MX$	Electric field force	Driving ions intercalation
**Lithiation of anode (alloy/de-alloy materials)**	Electrochemical: combination reaction	$e^{-}+M^{+}+X \rightarrow MX$	Strong reducibility of lithium ion	Driving ions migration
**Lithiation of anode (conversion materials)**	Electrochemical: replacement reaction	$zLi^{+}+ze^{-}+M_xN_y \rightarrow xM+Li_zN_y$	Strong reducibility of lithium ion	Driving ions migration

*M represents different metal materials * X represents different anode materials

* N represents O, P, S and N

Supplementary Table 3| Standard electrode potentials, Gibbs energies of formation and ionic radii of different metals [19,20]

		Au	Ag	Cu	Li	Pt	Cd
$E^o(M^+/M)$	Unit: V	1.498	0.799	0.342	-0.257	1.180	-0.403
$\Delta_f G^o(M_2S)$	Unit: KJ/mol	-219.5	-40.7	-86.2	-225.0	-76.2	-156.5
$R(M^+)$	Unit: pm	151	142	74	59	94	92

* M in the table represents different metals.

Supplementary References

1 Tang, J., Huo, Z., Brittman, S., Gao, H. & Yang, P. Solution-processed core-shell nanowires for efficient photovoltaic cells. *Nature Nanotech.* **6**, 568-572 (2011).

2 Beberwyck, B. J., Surendranath, Y. & Alivisatos, A. P. Cation Exchange: A Versatile Tool for Nanomaterials Synthesis. *J. Phys. Chem. C* **117**, 19759-19770 (2013).

3 Rivest, J. B. & Jain, P. K. Cation exchange on the nanoscale: an emerging technique for new material synthesis, device fabrication, and chemical sensing. *Chem. Soc. Rev.* **42**, 89-96 (2013).

4 Casu, A. *et al.* Cu2Se and Cu Nanocrystals as Local Sources of Copper in Thermally Activated In Situ Cation Exchange. *ACS Nano* **10**, 2406-2414 (2016).

5 Son, D. H., Hughes, S. M., Yin, Y. & Alivisatos, A. P. Cation exchange reactions in ionic nanocrystals. *Science* **306**, 1009-1012 (2004).

6 Goriparti, S. *et al.* Review on recent progress of nanostructured anode materials for Li-ion batteries. *J. Power Sources* **257**, 421-443 (2014).

7 Sadtler, B. *et al.* Selective facet reactivity during cation exchange in cadmium sulfide nanorods. *J. Am. Chem. Soc.* **131**, 5285-5293 (2009).

8 Zhang, D. *et al.* Phase-selective cation-exchange chemistry in sulfide nanowire systems. *J. Am. Chem. Soc.* **136**, 17430-17433 (2014).

9 Putnis, A. Electron diffraction study of phase transformations in copper sulfides. *Am. Mineral.* **62**, 107-114 (1977).

10 Mumme, W. G., Gable, R. W. & Petříček, V. The crystal structure of roxbyite, $Cu_{58}S_{32}$. *Can. Mineral.* **50**, 423-430 (2012).

11 Liu, Q. *et al.* Molten Au/Ge alloy migration in Ge nanowires. *Nano letters* **15**, 2809-2816 (2015).

12 Gao, P. *et al.* Electrically driven redox process in cerium oxides. *J. Am. Chem. Soc.* **132**, 4197-4201 (2010).

13 McDowell, M. T. *et al.* In situ observation of divergent phase transformations in individual sulfide nanocrystals. *Nano letters* **15**, 1264-1271 (2015).

14 Liu, X. H. *et al.* In situ atomic-scale imaging of electrochemical lithiation in silicon. *Nature nanotech.* **7**, 749-756 (2012).

15 Liu, X. H. *et al.* Ultrafast electrochemical lithiation of individual Si nanowire anodes. *Nano letters* **11**, 2251-2258 (2011).

16 Yuk, J. M. *et al.* Direct fabrication of zero-and one-dimensional metal nanocrystals by thermally assisted electromigration. *ACS nano* **4**, 2999-3004 (2010).

17 Slater, M. D., Kim, D., Lee, E. & Johnson, C. S. Sodium‐Ion Batteries. *Adv. Funct. Mater.* **23**, 947-958 (2013).

18 Wu, Z.-S. *et al.* Graphene anchored with Co3O4 nanoparticles as anode of lithium ion batteries with enhanced reversible capacity and cyclic performance. *ACS nano* **4**, 3187-3194 (2010).

19 Dean, J. Lange's Handbook of Chemistry, McGrawHill Book Co. *Inc.*, *New York* (1999).

20 Wang, L. *et al.* A quantum-chemical study on the discharge reaction mechanism of lithium-sulfur batteries. *J. Energ. Chem.* **22**, 72-77 (2013).
